# Evaluation of a Hydrodynamic Ozonation System for Gutta-Percha Disinfection and Structural Characterization

**DOI:** 10.1021/acsomega.5c08886

**Published:** 2026-02-09

**Authors:** Jessica Sthefanny Carvalho Souza, Pedro Augusto Laurindo Igreja Marrafa, Maycon Crispim Oliveira de Carvalho, Marcelo Fabiano Rodrigues, Bianca Akemi Kawata, Gislene Valdete Martins, Erick Gabriel Ribeiro dos Anjos, Fabio Roberto Passador, Adriana Barrinha Fernandes, Carlos José de Lima

**Affiliations:** † Postgraduate Program in Biomedical Engineering, Biomedical Engineering Institute, Universidade Anhembi Morumbi (UAM), São José dos Campos 12247-004, São Paulo, Brazil; ‡ Centro da Inovação, Tecnologia e Educação (CITE), São José dos Campos 12247-016, SP, Brasil; § Symrise Global Hair Care Center, Cotia 06715-725, SP Brazil; ∥ Coordination of Research and Technological Development from Instituto Nacional de Pesquisas Espaciais (COPDT/INPE), São José dos Campos 12201-970, São Paulo, Brazil; ⊥ Institute of Science and Technology, Federal University of São Paulo, São José dos Campos 04021-001, São Paulo, Brazil

## Abstract

Gutta-percha (GP), composed of the trans-isomer of polyisoprene, is widely used in endodontic obturation due to its favorable sealing properties. However, it is susceptible to contamination upon exposure to the clinical environment, and its thermoplastic nature precludes thermal sterilization. Conventional disinfection methods using sodium hypochlorite (NaOCl) or chlorhexidine (CHX) require strict control of concentration and exposure time to avoid structural damage. This study evaluated the efficacy of a hydrodynamic system using ozonated water for the inactivation of *Enterococcus faecalis*. Experimentally contaminated cones were exposed to a continuous flow of ozonated water for 5 and 7 min, followed by microbiological analysis. A seven-minute exposure (57.2 mg/L) resulted in complete bacterial inactivation, enabling further characterization of the biomaterial via FTIR spectroscopy, optical profilometry, scanning electron microscopy (SEM), and tensile strength testing. FTIR revealed subtle changes without compromising the polymeric backbone, while profilometry indicated a significant reduction in surface roughness. Mechanical testing showed preserved tensile strength and a reduction in elastic modulus, suggesting improved anatomical adaptation. SEM analysis did not reveal any relevant morphological alterations. These findings suggest that the hydrodynamic ozonated water system is an effective and safe alternative for disinfecting endodontic materials, with promising clinical applications.

## Graphical abstract

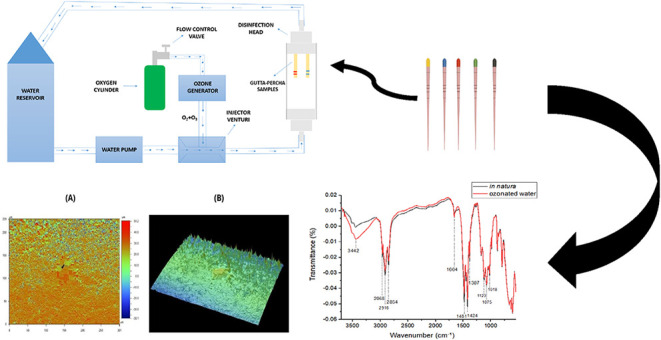

## 1 Introduction

Endodontic infections represent a significant challenge in clinical dental practice, affecting millions of individuals worldwide. These infections arise when microorganisms infiltrate the root canal system due to pulp necrosis, trauma, or failure of previous treatments.^1,2^ The complex three-dimensional anatomy of root canals,including isthmuses, lateral canals, and apical deltas creates favorable conditions for microbial colonization and biofilm formation.^3,4^

Endodontic treatment, commonly known as root canal therapy, aims to eliminate infection and prevent reinfection of the intricately structured root canal system.^5^ Success in endodontic therapy depends on several critical factors, including precise canal shaping, effective disinfection protocols, and achieving a complete seal through three-dimensional obturation.^6^

One of the primary causes of endodontic treatment failure is the persistence of certain bacteria within the root canal system, with *Enterococcus faecalis* being among the most resistant to conventional disinfection methods. This resistance allows the infection to persist both within and beyond the canal. *E. faecalis* is a Gram-positive anaerobic coccus commonly found in the oral cavity, gastrointestinal tract, and vaginal region, thriving in nutrient-rich, low-oxygen environments. Studies have shown that this bacterium is more prevalent in cases of endodontic failure than in primary infections and has been identified in up to 90% of cases involving post-treatment pain and infection.^7^

Gutta-percha cones are widely used in root canal obturation due to their favorable properties, including biocompatibility, resistance to deformation, and radiopacity. However, they can become contaminated through contact with gloves, aerosols during clinical handling, or improper storage. Since infection control is essential for successful endodontic therapy, disinfecting the cones prior to insertion into the canal is crucial. Conventional sterilization methods using moist or dry heat cannot be applied without compromising the material’s structural integrity, making chemical disinfection the most viable alternative for ensuring microbiological safety.^8^

The composition of commercially available gutta-percha cones typically consists of approximately 20% gutta-percha polymer, composed of a trans-polyisoprene matrix; 66% zinc oxide, which serves as a filler agent; 11% heavy metal sulfates, responsible for radiopacity; and around 3% waxes or resins, which act as plasticizers. Gutta-percha is derived from latex and is an isomer of rubber known as trans-polyisoprene, exhibiting the typical characteristics of a partially crystalline viscoelastic polymeric material.^9^

The literature highlights significant variability in the composition of gutta-percha cones among different manufacturers. For instance, the proportion of gutta-percha polymer may vary depending on the brand.^10^ Additionally, the presence of filler agents such as barium sulfate is not consistent across products; in some cases, this component is absent, which can directly affect properties such as radiopacity and sealing ability.^11^ Although the composition of gutta-percha cones is considered relatively standardized, these intermanufacturer variations may impact clinical performance, underscoring the importance of careful material selection based on the specific requirements of each endodontic procedure.

Contamination of gutta-percha (GP) cones by *E. faecalis* is a significant concern in endodontics, as it can lead to therapeutic failure. Studies have shown that surface properties of the cones, such as roughness and waviness, promote bacterial adhesion,particularly in deeper regions like the valleys of the cones.^12^

Disinfectant agents such as sodium hypochlorite (NaOCl), chlorhexidine (CHX), and peracetic acid (PAA) are commonly used for rapid disinfection of gutta-percha cones in clinical practice. The ideal disinfectant must effectively eliminate microorganisms without compromising the structural integrity of the material.^13^

Current applications of ozone therapy in dentistry include biofilm reduction, preventive and restorative dentistry, periodontics, endodontics, oral pathology and surgery, implantology, wound healing, decontamination of dental materials, treatment of dentin hypersensitivity, pain management, and temporomandibular disorders.^14^

Ozone exhibits potent antimicrobial properties, acting effectively against bacteria, fungi, and viruses without causing harm to human cells. Its efficacy is particularly notable against antibiotic-resistant microorganisms, making it a valuable tool in infection management.^14^ Furthermore, studies have demonstrated its effectiveness in postoperative contexts, with significant pain reduction and enhanced wound healing, as observed in third molar extractions.^15^ Clinically, ozone therapy is characterized by high biocompatibility and minimal side effects, promoting greater patient comfort and recovery.

In clinical practice, ozone can be applied in gaseous form, ozonated water, or ozonated oil. It is used in nonsurgical periodontal treatments, in the control of cariogenic pathogens, and as an adjunct in scaling, root planing, and gingival curettage. Ozonated water stands out for its biocompatibility with human oral cells, low risk of adverse effects, and efficacy against resistant microorganisms, whereas gaseous ozone requires specific precautions to avoid inhalation. Due to its noninvasive, safe, and efficient nature, ozone therapy represents a significant advancement in dentistry, enhancing patient experience and improving clinical outcomes when applied in controlled doses.^13^

The study demonstrated that ozone in aqueous solution exhibits antimicrobial efficacy comparable to that of sodium hypochlorite (NaOCl) and chlorhexidine (CHX) against *E. faecalis* biofilms. When tested against other microbial species, including *Streptococcus mutans* and *Candida albicans*, aqueous ozone also resulted in a significant reduction in biofilm formation following irrigation.^16^

However, being recognized for its strong oxidative capacity, which surpasses that of hydrogen peroxide,ozone is able to react with a broad range of biological materials. Studies have shown that it can induce oxidative damage to lipids, proteins, and nucleic acids, as well as to more complex substrates such as human hair fibers and tattoo pigments.^17–21^ This remarkable reactivity not only underpins its potential applications in sanitation and therapeutic contexts but also emphasizes the risks of structural degradation when it interacts with biological tissues.

Previous research has shown that evaluated the efficacy of ozonated sunflower oil in eliminating biofilms of antibiotic-resistant *E. faecalis*, comparing its performance to gaseous ozone. The experiments were carried out on contaminated composite resin discs subjected to different treatment protocols. The results showed that both ozone forms,gaseous and ozonated oil,led to a significant reduction in colony-forming units (CFU/mL) compared to control groups. Notably, the protocol involving ozonated sunflower oil for 10 min demonstrated the highest antimicrobial efficacy, highlighting it as a viable and less invasive alternative for managing endodontic infections caused by resistant microorganisms.^22^

Given the growing need for new strategies to control microbial infections, the use of disinfectant agents such as ozonated water emerges as a promising, effective, and safe alternative. In this context, the present study aims to evaluate the efficacy of a hydrodynamic system using ozonated water for the inactivation of *E. faecalis*.

The objective of this study is to optimize the disinfection of gutta-percha cones while preserving their structural integrity, thereby contributing to the safety and success of endodontic treatments. In addition to microbiological analysis, the biomaterial was characterized after the ozonation process using techniques such as Fourier Transform Infrared Spectroscopy (FTIR), Optical Profilometry, Mechanical Tensile Testing, and Scanning Electron Microscopy (SEM), allowing for a detailed evaluation of potential physical and chemical changes resulting from the disinfection procedure.

## 2 Results

### 2.1 Determination of Water Flow Velocity

The average velocity of ozonated water in the hydrodynamic system was calculated based on the volumetric flow rate equation *Q* = *A.V*, where Q is the flow rate, *A* is the cross-sectional area of the tube, and *V*_m_ is the average fluid velocity. For a tube with an internal diameter of 1.4 cm, the calculated cross-sectional area was 1.54 cm^2^. The measured flow rate was 22 cm^3^/s. Substituting these values into the equation, the average velocity of the ozonated water was estimated to be approximately 14.3 cm/s.

### 2.2 O_3_ Concentration Curve Dissolved in Water

Based on the exposure duration established in prior research,^23,24^ an ozone concentration of approximately 4.5 mg L^–1^ was achieved within 600 s (10 min). The concentration profile showed an initial rapid increase, followed by a more gradual rise, suggesting that the system approaches stabilization over time (Figure 1). The experimental data were well described by a double exponential model, *c*(*t*) = −2.54·e^–*t*/0.82^ + 0.62·e^–*t*/6.68^ + 1.81, which yielded a coefficient of determination of *R* ^2^ = 0.99, indicating a strong correlation between exposure time and ozone concentration.

**Figure 1:**
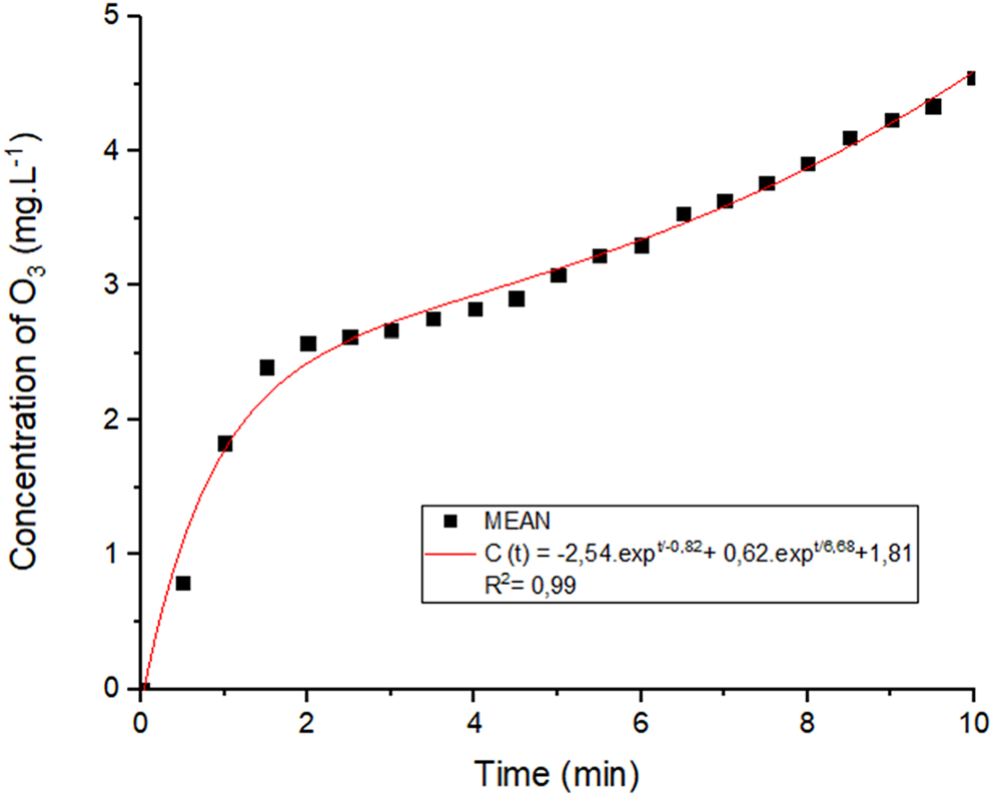
O_3_ concentration curve dissolved in water at 18 °C. Source: Author’s own work.

This mathematical model was selected for its capacity to capture the nonlinear dynamics of ozone concentration over time, providing an empirical fit to the experimental data and accurately representing both the growth and stabilization phases of the ozonation process. Moreover, the model allows for the estimation of ozone concentration as a function of time, which may prove valuable in future studies for determining optimal exposure conditions for various applications.

### 2.3 Microbiological Analysis Results

Initially, assays were conducted with an exposure time of 5 min. However, in half of the samples exposed to the hydrodynamic system with ozonated water, turbidity was observed in the culture medium after 24 h, indicating bacterial growth (Table 1). Due to this limitation, a new exposure time of 7 min was established.

**Table 1:** Determination of the Presence or Absence of Microbial Growth in GP Samples across Different Experimental Groups

samples	24 h	48 h	7 days
positive control	15^a^/15^a^	15^a^/15^b^	15^a^/15^b^
negative control	0^a^/15^b^	0^a^/15^b^	0^a^/15^b^
O_3__5 min	7/15^b^	7^a^/15^b^	7^a^/15^b^
O_3__7 min	0^a^/15^b^	0^a^/15^b^	0^a^/15^b^

a Number of samples showing microbial growth.

b Total number of samples (*n* = 15).

The negative control tubes remained clear, indicating the absence of bacterial growth in the untreated cones. In the positive control, turbidity was observed in the medium, confirming bacterial growth and demonstrating that *E. faecalis* remained viable throughout the experiment.

In the tubes containing cones subjected to experimental infection and treated with the hydrodynamic system for seven minutes, the medium remained clear, indicating complete elimination of the bacterium. These results suggest that the hydrodynamic ozonation system was effective in disinfecting GP cones, eliminating *E. faecalis* and preventing bacterial proliferation.

### 2.4 Quantitative Microbiological Analysis

The GP samples were initially inoculated with an *E. faecalis* suspension at a concentration of 10^6^ CFU/mL. Microbial recovery assays were then conducted to quantify the bacterial load remaining adherent to the material surface. Quantitative analysis revealed an initial adherent bacterial concentration of 10^4^ CFU/mL. Following a 7 min ozonation treatment, no viable bacterial colonies were detected, indicating a reduction to 10 °CFU/mL. This result corresponds to a 4-log reduction in the microbial population, equating to a 99.99% decrease in viable bacteria (Table 2).

**Table 2:** Quantitative Evaluation of *E. faecalis* Reduction after Ozonation Treatment

experimental stage	*t*_0_	*t*_7_
microbial recovery assays	10^4^	10^0^
log reduction		4
percent reduction (%)		99,99

### 2.5 Fourier Transform Infrared Spectroscopy (FTIR)

FTIR spectroscopy was employed to investigate potential chemical modifications in gutta-percha cones following exposure to ozonated water for 7 min. Spectra were obtained for both untreated (in natura, black) and ozonated (red) samples, indicating no significant changes in the chemical composition of the polymeric matrix (Figure 2).

**Figure 2:**
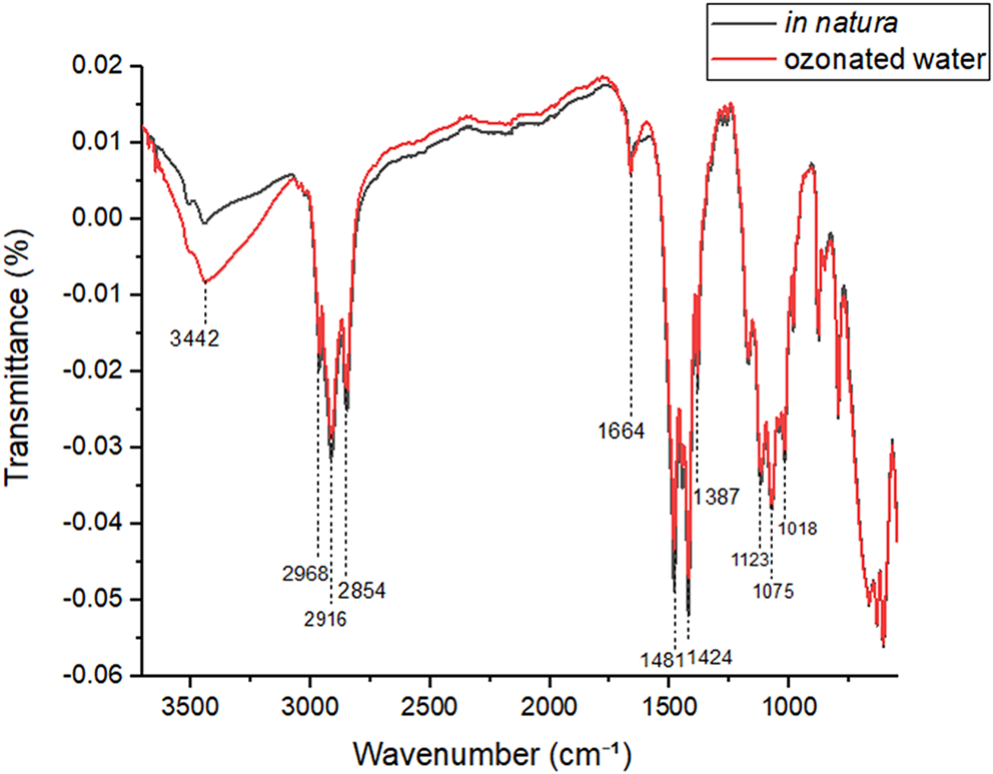
FTIR spectra of gutta-percha (GP) cones in their untreated state (in natura) and after exposure to ozonated water (O_3_). Source: Author’s own work.

Subtle alterations were observed in specific regions, likely restricted to the material’s surface: at 3442 cm^–1^, a slight increase in intensity was noted, consistent with the presence of hydroxyl (−OH) groups. At 1664 cm^–1^, a minor increase in the band associated with C=O stretching was detected, which may suggest the onset of oxidation.

### 2.6 Analysis of Data from Optical Profilometry

The analysis of data obtained through optical profilometry (Table 3) revealed a significant impact of ozonated water protocols on the surface roughness of gutta-percha (GP) cones. Statistical evaluation of the average roughness (Ra) showed a notable difference between the experimental groups, with a percentage variation of approximately 45%.

**Table 3:** Statistical Analysis of Ra Values for Gutta-Percha (GP)^a^

parameter	in natura (μm)	O_3_(μm)
mean	3.52	1.94
sample Size	9	9
standard Deviation (SD)	0.25	0.24
*p*-value	<0.0001	

a Source: Author’s own work.

The two-tailed *p*-value was less than 0.0001, indicating an extremely significant result. In a two-tailed test, the probability that the observed difference between groups occurred by chance is assessed by considering deviations in both directions from the expected mean (higher or lower).

In Figure 3, corresponding to the in natura group, both two-dimensional (A) and three-dimensional (B) surface representations are shown. Image 3A reveals a heterogeneous topographic distribution, with height variations ranging from approximately −20.1 μm to +38.3 μm. This wide range indicates a rough surface, characterized by peaks and depressions distributed across the sample. The three-dimensional view in Image 3B confirms the presence of an irregular texture, with centrally accentuated elevations, possibly indicative of localized structural modifications.

**Figure 3:**
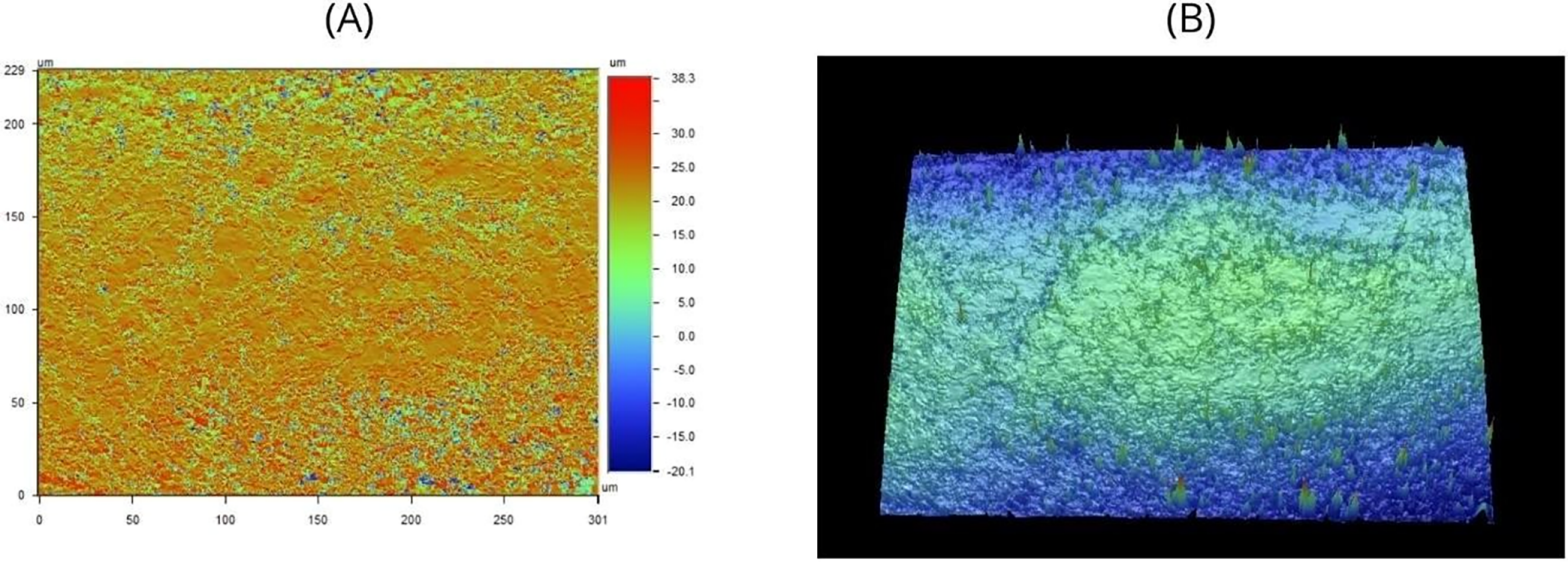
Representation of gutta-percha in natura samples in optical profilometry. Panel A shows a planar view with height mapping indicated by the adjacent color scale, while Panel B presents a three-dimensional view of the sample surface. Source: Author’s own work.

Following exposure to ozonated water, Figure 4 shows visible changes in the surface microtopography. The vertical variation scale expanded to a range between – 38.1 μm and +50.2 μm, suggesting an increase in the amplitude of surface irregularities (Figure 4A). Additionally, a centrally located region with a geometric and markedly altered pattern is observed, suggesting surface removal or reorganization of the polymeric material, likely caused by the oxidative action of ozone.

**Figure 4:**
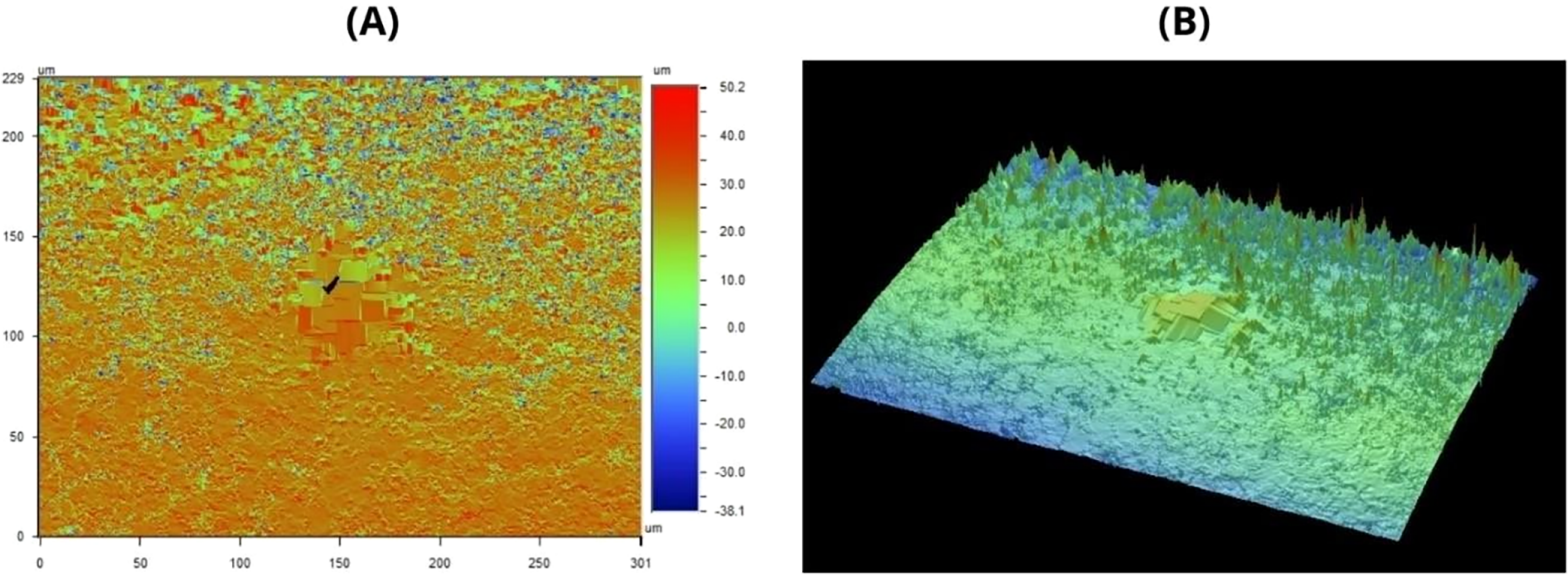
Representation of gutta-percha samples following exposure to the hydrodynamic system, as observed through optical profilometry: (A) surface data; (B) interactive three-dimensional visualization. Source: Author’s own work.

The 3D reconstruction (Figure 4B) confirms these changes, showing more prominent peaks and an irregular distribution of surface roughness, particularly along the edges of the sample. These findings suggest that ozonated water induces structural modifications on the surface of gutta-percha, possibly due to partial degradation of the polymeric matrix or superficial reorganization.

### 2.7 Tensile Strength and Elastic Modulus Testing

Figure 5 presents the stress–strain curves obtained from tensile tests conducted on gutta-percha cones under two conditions: untreated (black curve) and after exposure to ozonated water (red curve).

**Figure 5:**
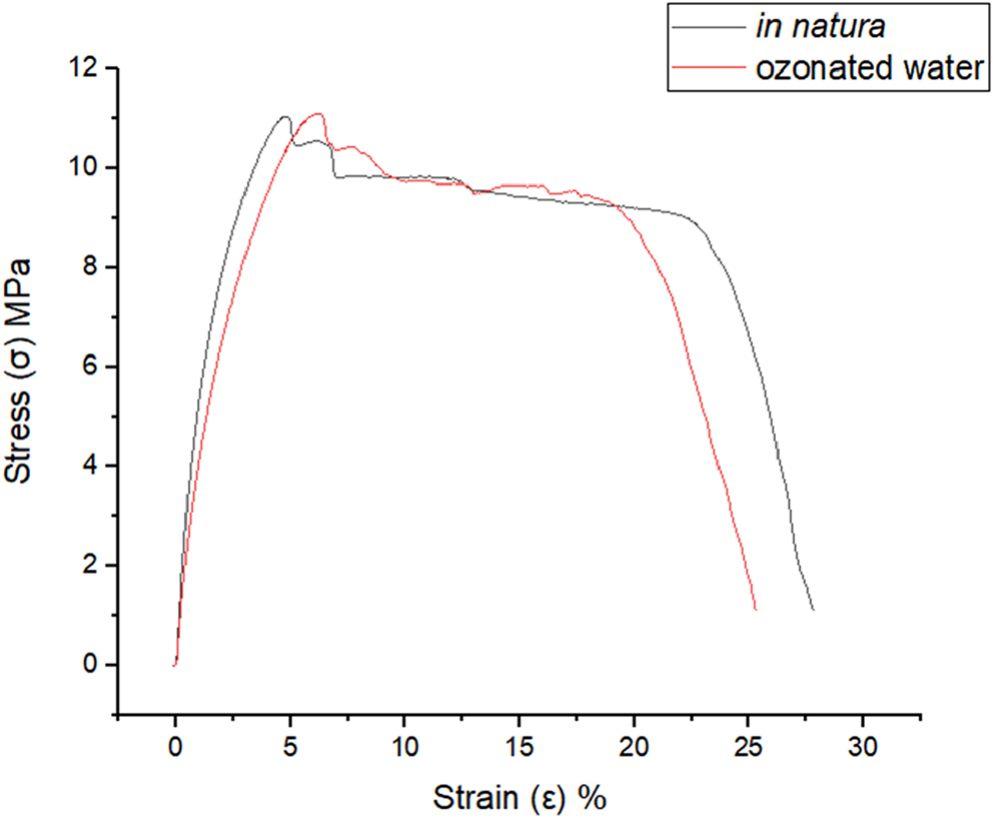
Results of Tensile Strength and Elastic Modulus Tests. Source: Author’s own work.

The data presented in Table 4 correspond to the peak stress values, representing the material’s tensile strength limit obtained for each group. A total of six samples were analyzed in this evaluation. Exposure to ozonated water for 7 min resulted in an average reduction of approximately 0.84% in tensile strength compared to the untreated samples. Statistical analysis revealed no statistically significant difference between the group means. These findings indicate that the ozone treatment protocol did not produce a significant effect on the measured variable when compared to the control condition.

**Table 4:** Statistical Analysis of Tensile Strength Tests^a^

parameter	in natura (MPa)	O3 (MPa)
mean	9.85	9.77
standard deviation (SD)	1.06	1.42
*p*-value	0.9108	

a Source: Author’s own work.

Table 5 presents a comparison between the in natura group and the group exposed to ozonated water for 7 min. A total of six samples were analyzed in this comparison. Regarding the elastic modulus, a reduction of approximately 27.01% was observed following processing in the hydrodynamic system. These findings suggest that ozone treatment led to a significant decrease in the elastic modulus compared to the in natura group.

**Table 5:** Statistical Analysis of Elastic Modulus Results^a^

parameter	in natura (MPa)	O3 (MPa)
mean	742.5	541.67
standard deviation (SD)	60.2	31.61
*p*-value	0.0002	

a Source: Author’s own work.

### 2.8 Scanning Electron Microscopy (SEM)

Morphological analyses of the untreated gutta-percha cones were performed using Scanning Electron Microscopy (SEM), as shown in Figure 6. At lower magnification (Figure 6A), the surface of the cones appeared smooth and homogeneous, with few visible irregularities. As magnification increased (Figure 6B), the presence of micropores and structural heterogeneities distributed across the surface became evident. In the most magnification (Figure 6C), the pores were more clearly defined compared to those observed in Figure 6A.

**Figure 6:**
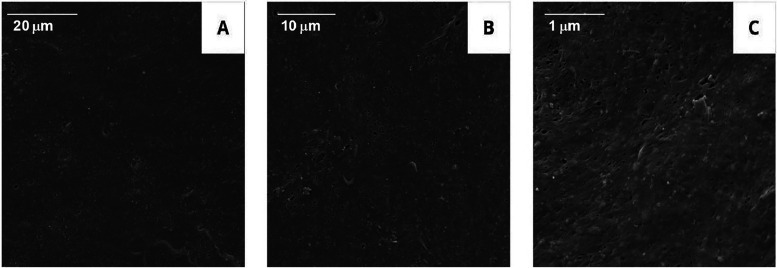
SEM micrographs of untreated gutta-percha cones. (A) Scale: 20 μm; (B) Scale: 10 μm; (C) Scale: 1 μm. Source: Author’s own work.

A comparison between the images of samples exposed to ozonated water for 7 min (Figure 7) and the untreated samples revealed no relevant morphological differences on the material’s surface between the groups. Scanning electron micrographs from both groups showed homogeneous surfaces with discrete porosity and no visible fissures. The surface topography remained visually similar across both conditions, indicating that the oxidative action of ozone, under the tested conditions, was not sufficient to induce detectable structural changes.

**Figure 7:**
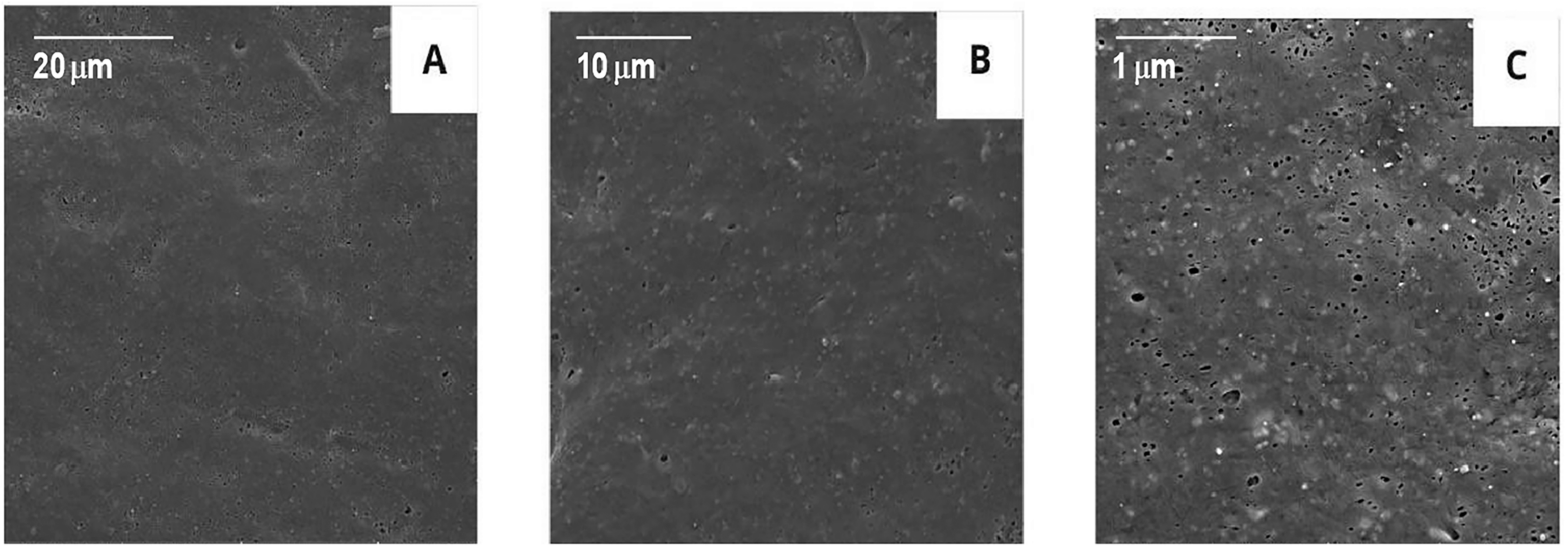
Scanning Electron Microscopy (SEM) images of gutta-percha cones following exposure to ozonated water in a hydrodynamic system for 7 min. (A) Scale: 20 μm; (B) Scale: 10 μm; (C) Scale: 1 μm. Source: Author’s own work.

## 3 Discussion

The microbiological analysis demonstrated the effectiveness of the hydrodynamic system with ozone in inactivating *E. faecalis* in contaminated gutta-percha cones. The results indicated that a 7 min exposure to ozonated water, at a concentration of 57.2 mg/L generated by the ozone device, was sufficient to inactivate the microorganism, whereas shorter exposure times, such as 5 min, resulted in bacterial growth.

When comparing these findings with previous research, the effects of ozone and ultrasound on *S. mutans*, a key etiological agent in dental caries, were evaluated using scanning electron microscopy and atomic force microscopy. In addition, microbial viability and the formation of reactive oxygen species (ROS) were assessed following the application of both techniques. The results demonstrated that, although both methods exhibited microbicidal effects and induced morphological changes, ozone was more efficient, significantly increasing ROS production and achieving bacterial inactivation four times faster, with 12 times less energy consumption compared to ultrasound.^25^

These findings were also compared with previous studies employing the same hydrodynamic system for in vitro disinfection of biomaterials, such as Human Amniotic Membrane (HAM). Methodologically, both studies standardized the inoculation of *E. faecalis* and *Staphylococcus aureus* using serial dilution techniques and evaluated outcomes based on bacterial growth in appropriate culture media. In the present study, 7 min of ozonation (57.2 mg/L) were required for gutta-percha disinfection, whereas previous work reported a need for 15 min (62 mg/L) to achieve disinfection of HAM.^26^

The study by Botelho et al. investigated the sterilization of MAH using a hydrodynamic system. The authors reported that exposure to ozonated water for 40 min (62 mg/L) effectively eliminated *S. aureus*, *E. coli*, *C. albicans*, *S. epidermidis*, and *Clostridium sporogenes spores*. Both Botelho et al. and Awoyama et al. (2022) explored the application of the hydrodynamic system with ozonated water for the disinfection and sterilization of biological tissues, demonstrating its versatility as an antimicrobial agent.^27^

Based on the results obtained and comparison with previous studies, it can be concluded that the hydrodynamic system with ozonated water, as well as ozone itself, represents an effective strategy for disinfecting gutta-percha cones contaminated with *E. faecalis*. This approach was capable of promoting microbial inactivation in significantly shorter exposure times than those reported for other biomaterials. The observed efficacy after just 7 min of exposure reinforces the potential of ozone as a versatile antimicrobial agent, with rapid and consistent action, in alignment with literature evidence highlighting its superiority over other techniques, such as ultrasound. These findings contribute to expanding the clinical application prospects of both the hydrodynamic system and ozone, particularly in the endodontic context, where minimizing the risk of cross-contamination is essential for therapeutic success.

Fourier Transform Infrared (FTIR) Spectroscopy analyses are used to assess the chemical composition and structural changes in gutta-percha cones. The antimicrobial effects of gutta-percha incorporated with chlorhexidine (CHX) were previously evaluated using FTIR spectroscopy. In the study, 80 conventional gutta-percha cones were used, with 10 cones disinfected by immersion in a 2% chlorhexidine solution for 10 min and stored in sterile distilled water for 24 h.^28^

The chemical incorporation of CHX into the gutta-percha cones was analyzed via FTIR, and the antimicrobial effect was assessed using a microbial sensitivity test. FTIR results indicated that chemical incorporation of chlorhexidine occurred after immersion in the 2% solution. Prior to immersion, the gutta-percha cones exhibited a characteristic C=C stretching vibration band at 1482 cm^–1^, which disappeared following CHX incorporation, suggesting a chemical interaction between CHX and the gutta-percha material. After immersion, the CHX-treated cones displayed a new vibration at 1636 cm^–1^, indicating that chlorhexidine was integrated into the material. This confirms that CHX was not merely adsorbed onto the surface but chemically bonded to the cone structure, evidencing changes detectable by spectroscopy. These results demonstrated significant chemical modification of the gutta-percha cones following treatment with the CHX solution.

In contrast, exposure of gutta-percha cones to the hydrodynamic system resulted in very subtle spectral changes, which may be attributed to mild surface oxidation induced by ozonated water. This suggests that the protocol did not compromise the main polymeric structure, trans-1,4-polyisoprene. The changes detected by FTIR fall within the detection threshold for surface-level modifications, which may be of interest from the perspective of light surface functionalization without affecting the material’s overall mechanical properties.

The evaluation of the effects of chemical disinfection and exposure to ozonated water on the surface roughness of gutta-percha cones revealed significant differences among the methods assessed. The use of chemical solutions such as 2.5% sodium hypochlorite (NaOCl) for 10 min and 2% chlorhexidine (CHX) for 5 min has been shown to result in a statistically significant reduction in surface roughness (*p* < 0.01), suggesting a smoothing effect due to the dissolution of microscopic irregularities. In contrast, 5.25% NaOCl applied for only 1 min showed a smaller reduction in roughness, which may be attributed to the shorter exposure time.^29^

Data from the present study, obtained through optical profilometry, demonstrate that exposure to ozonated water for 7 min also led to a significant reduction in the average roughness (Ra) of the cones. In a hydrodynamic system, moving water possesses kinetic energy, expressed by the equation Ec = (m·v^2^)/2, which is directly proportional to the fluid’s mass and the square of its velocity. During cone exposure to the flow, the fluid’s kinetic energy generates shear forces on the material’s surface, progressively reducing microirregularities. This effect is intensified in areas with greater surface relief, promoting a leveling of the material’s topography.

Therefore, while previous studies suggest that chemical solutions act by dissolving surface irregularities, the present research shows that ozonated water can achieve a similar effect without the use of potentially aggressive chemical compounds.

A reduction in the surface roughness of gutta-percha cones may have positive effects on endodontic sealing. Smoother surfaces promote better adaptation of the cone within the root canal, minimizing the formation of voids and, consequently, reducing the risk of microleakage. Low surface roughness can contribute to a more stable interface between the cone and the sealer, enhancing its sealing capacity. Additionally, less irregular surfaces are less prone to microbial and biofilm retention, representing an added factor for clinical biosafety.

However, excessive immersion times in disinfectant solutions may induce significant morphological changes on the gutta-percha surface, compromising the cones’ adaptability to the root canal and, therefore, the effectiveness of obturation. It is thus essential to establish disinfection protocols that balance biosafety with the preservation of material properties. As a proposal for future studies, it is suggested to evaluate the adhesion of different endodontic sealers to gutta-percha following various disinfection protocols, in order to identify which conditions yield better results in terms of sealing and obturation stability.

Regarding mechanical properties, the impact of sodium hypochlorite (NaOCl) disinfection on the mechanical behavior of gutta-percha cones has been evaluated. Two concentrations (2.5% and 5.25%) and different immersion times (1, 5, and 10 min) were tested. The results showed that tensile strength was not significantly affected in any of the groups; however, the elastic modulus was significantly reduced following exposure to NaOCl, particularly at higher concentrations.^30^

The tensile strength limit indicated that exposure of gutta-percha cones to ozone, under the experimental conditions adopted, did not compromise the mechanical integrity of the material in terms of its ultimate strength. This result is relevant, as it supports the feasibility of using ozone as an auxiliary agent in disinfection protocols without impairing the strength of the obturation material. The relative reduction calculation revealed an approximate 0.84% decrease in maximum tensile strength in the ozone-treated group compared to the control group,a minimal and clinically irrelevant variation.

The observed reduction of approximately 27% in the elastic modulus following treatment with ozonated water may represent a clinically beneficial modification. A lower elastic modulus indicates greater material flexibility, which may enhance its adaptation to the anatomical irregularities of root canal structures, contributing to more effective sealing. Moreover, increased flexibility may reduce the risk of fractures and improve the material’s performance under mechanical stress.^31^

Improved adaptation to the anatomical irregularities of the root canal system may facilitate the filling of curved regions, lateral branches, or complex morphologies, reducing the presence of unfilled spaces and contributing to a more precise and complete obturation. This promotes a more effective and continuous seal, which is essential for preventing microbial infiltration and therapeutic failure. Accordingly, the results suggest that exposure of gutta-percha to ozone may beneficially alter its mechanical properties, enhancing its clinical performance in specific scenarios.

In summary, the results indicate that disinfection of gutta-percha cones with ozonated water did not compromise their tensile strength, preserving the essential mechanical integrity of the obturation material. Although a reduction in the elastic modulus was observed, this effect may represent a favorable modification, as increased elasticity tends to improve the cone’s adaptation to the anatomical irregularities of the root canal, contributing to a more precise and effective seal. Compared to the study by Bellido-Guzmán et al.,^30^ in which NaOCl also significantly reduced the elastic modulus, ozone offers efficient antimicrobial action without the need for chemical additives, thereby reducing potential risks associated with residues or deleterious alterations to the material.

The SEM analyses conducted in this study demonstrated the absence of relevant morphological differences between the evaluated groups. The images revealed homogeneous surfaces in both groups, with discrete porosities and no evidence of fissures or significant structural degradation, even under higher magnifications. Surface topography remained visually similar, suggesting that, under the tested conditions, the oxidative action of ozone was not sufficient to induce structural changes detectable by SEM.

These findings are comparable to previous results in which the apical third of 60 standardized gutta-percha cones from six different manufacturers was analyzed. Although most cones exhibited smooth and uniform surfaces, some showed morphological irregularities, such as pronounced protrusions containing crystalline particles, either free or embedded,features also observed in the micrographs obtained in the present study. Thus, it becomes evident that the craters identified in the cones, both before and after exposure to ozonated water, may be related to the manufacturing process. Furthermore, the results reinforce the efficacy of ozonated water, indicating that its application does not compromise the structural integrity of the material.^32^

In contrast to previous findings, the action of 1% and 5.25% NaOCl solutions, with or without surfactant, on the disinfection of gutta-percha cones contaminated with *E. faecalis* was associated with surface modifications observed under scanning electron microscopy, including the formation of sodium chloride crystals. These alterations were less evident when the 1% NaOCl solution was combined with a surfactant. These results demonstrate that, despite its antimicrobial efficacy, the use of NaOCl may lead to undesirable structural changes on the surface of gutta-percha.^7^

In conclusion, the SEM analyses performed in this study showed that exposure of gutta-percha cones to ozonated water did not cause relevant morphological changes, preserving homogeneous surfaces without fissures or significant structural degradation. The minor irregularities observed appear to be related to the manufacturing process, corroborating previous findings by Goldberg et al.^32^ These results indicate that the oxidative action of ozone, under the tested conditions, is capable of promoting effective disinfection without compromising the structural integrity of the material. In contrast, studies involving NaOCl, such as that by Vitali et al.,^7^ highlight that although effective, hypochlorite may induce undesirable surface modifications, including the deposition of sodium chloride crystals. Therefore, ozonated water emerges as a promising alternative for gutta-percha cone decontamination, combining antimicrobial efficacy with the preservation of the material’s morphological characteristics.

In a real clinical scenario, the exposure of gutta-percha cones to ozonated water may offer an additional advantage related to fluid dynamics within the root canal system. Following the ozonation process, it is possible that a thin residual film of ozonated water remains adhered to the surface of the cone. This film may flow with reduced resistance through the dentinal tubules,microscopic structures within the dentin that play a crucial role in the tooth’s microarchitecture and function.^33^ This condition favors deeper penetration of the disinfecting agent, dissolved ozone, into microstructures of the canal system, enhancing the inactivation of residual microorganisms in hard-to-reach areas.

The use of ozonated water in dentistry also offers environmental benefits, serving as a natural and eco-friendly alternative to traditional chemical disinfectants. Unlike many conventional dental chemicals, ozone (O_3_) rapidly decomposes into oxygen (O_2_) after use, leaving no harmful residues or environmental pollutants. This prevents the release of toxic substances into wastewater and the environment, making ozone a sustainable option for sterilization and disinfection in dental practices.^34^

In an integrated manner, the present study stands out for its multidisciplinary approach and methodological rigor in evaluating the disinfection of GP cones using a hydrodynamic system with ozonated water. This system provided an ozone concentration of 57.2 mL/L at the generator outlet and reached a dissolved ozone concentration of 4.5 mg·L^–1^ in the water over a 10 min period. The controlled exposure of the cones for 7 min promoted contact of the agent with the material surface, optimizing contact efficiency compared to static immersion methods and facilitating microbial inactivation.

The use of a standardized *E. faecalis* ATCC 29212 monoculture, calibrated to 10^6^ CFU/mL, ensured greater experimental accuracy and reproducibility of the results, considering the clinical relevance of this microorganism, which is widely associated with endodontic treatment failures. Beyond microbiological validation, obtained through qualitative and quantitative analyses, the study incorporated complementary techniques, including FTIR spectroscopy, optical profilometry, tensile testing, and SEM, which confirmed the preservation of the material’s structural, surface, and mechanical properties after exposure to ozonated water.

Thus, the present research validates the use of the hydrodynamic system as an effective and safe approach for the decontamination of GP cones, highlighting its potential as a sustainable alternative to conventional chemical methods by providing efficient disinfection without compromising the integrity of the biomaterial.

## 4 Conclusions

This in vitro study demonstrated that the use of a hydrodynamic system with ozonated water is an effective and safe alternative for the disinfection of gutta-percha cones contaminated with *E. faecalis*. A 7 min exposure (57.2 mg/L) was sufficient to achieve complete bacterial inactivation without significantly compromising the chemical or mechanical properties of the biomaterial. FTIR analysis indicated no significant changes in the chemical composition of the polymeric matrix, while optical profilometry revealed a significant reduction in surface roughness, which may enhance anatomical adaptation within the root canal.

Although a reduction in the elastic modulus was observed following ozonation, this change may positively contribute to the material’s flexibility without compromising its tensile strength. Additionally, SEM images confirmed the preservation of the morphological integrity of the cone surfaces. Taken together, these findings suggest that the hydrodynamic system with ozonated water is a promising approach that could be incorporated into clinical practice as a sustainable alternative to traditional chemical disinfectants.

## 5 Methods

This *in vitro* study aimed to assess the efficacy of a hydrodynamic ozonation system for disinfecting gutta-percha cones contaminated with *E. faecalis*. The investigation combined microbiological analysis with an evaluation of the chemical and mechanical properties of the cones following a 7 min ozone exposure. The experimental setup utilized 98 gutta-percha cones (Figure 8) and was constructed exclusively with components resistant to oxidative degradation by ozone. These included a 500 mL acrylic water reservoir, flexible silicone tubing for fluid transport, a centrifugal pump, and a polymer-based Venturi injector. To maintain aseptic conditions, all system elements, with the exception of the pump and Venturi valve, were sterilized in an autoclave prior to experimentation.

**Figure 8:**
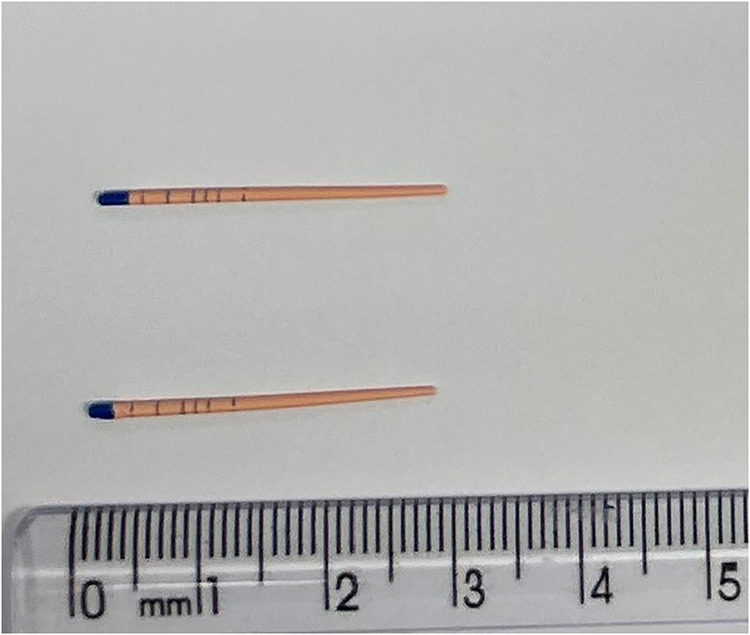
Gutta-percha cone used in the experimental procedures. Source: Author’s own work.

### 5.1 Experimental Inoculation of Samples and Disinfection Protocol

The system consisted of a 500 mL water reservoir connected to a centrifugal pump. A Venturi injector was attached to the pump outlet, with a third inlet for ozone gas, enabling dynamic mixing with water. The fluid was directed to a tubular head via silicone tubing, where the gutta-percha samples were positioned for disinfection (Figure 8). The flow velocity and ozone’s oxidative action were expected to remove and oxidize organic material deposited on the sample surfaces.

Experiments were conducted in a climate-controlled environment at 21 °C. An ozone generator (MS3G, MS Ltd.a, Brazil) was set to a concentration of 57.2 mg/L, with oxygen gas flow adjusted to 0.250 L/min. Initially, the system was activated without samples to allow complete ozonation of the water and internal disinfection of the system, requiring 10 min to inactivate any residual microorganisms.

Two exposure times were tested: 5 and 7 min. The gutta-percha cones were placed in a custom-made Teflon sample holder capable of accommodating up to eight cones simultaneously. The cones were experimentally contaminated with *E. faecalis* (ATCC 29212), cultured for 24 h at 37 °C in Tryptic Soy Agar (KASVI Platinum), and suspended in sterile saline at a final concentration of 10^6^ CFU/mL, adjusted using the McFarland nephelometric method.

The final concentration of *E. faecalis* was adjusted to 10^6^ CFU/mL in sterile saline solution through serial dilution, based on the nephelometric method (McFarland scale). The disinfection chamber containing the gutta-percha cones was then placed in a 40 mL beaker, where it remained for 30 min.

After the designated bacterial inoculation period, samples were collected in triplicate for both positive and negative controls. The positive control consisted of cones previously contaminated with *E. faecalis* (ATCC 29212), while the negative control comprised cones in their original, sterile condition directly from the manufacturer’s sealed packaging. The sample holder containing the contaminated gutta-percha cones was inserted into a 23 mm diameter acrylic tube, ensuring direct contact with the ozonated water (Figure 9). It is important to note that the samples were not expected to dry prior to the treatment phase Figure 10.

**Figure 9:**
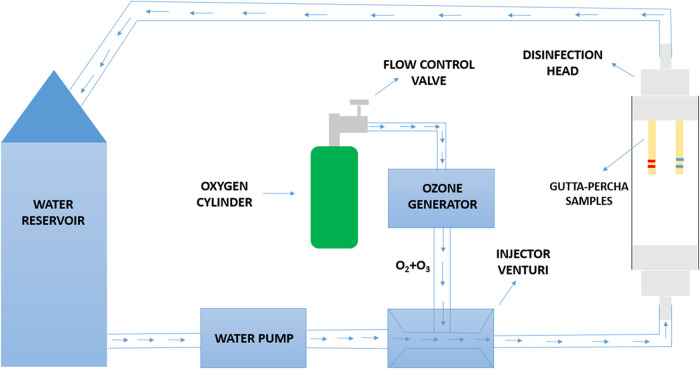
Schematic representation of the system used for gutta-percha disinfection with ozonated water via a hydrodynamic setup. The samples, consisting of eight units, were internally fixed within the tubular head and exposed to the dynamic flow of ozonated fluid. Source: Author’s own work.

**Figure 10:**
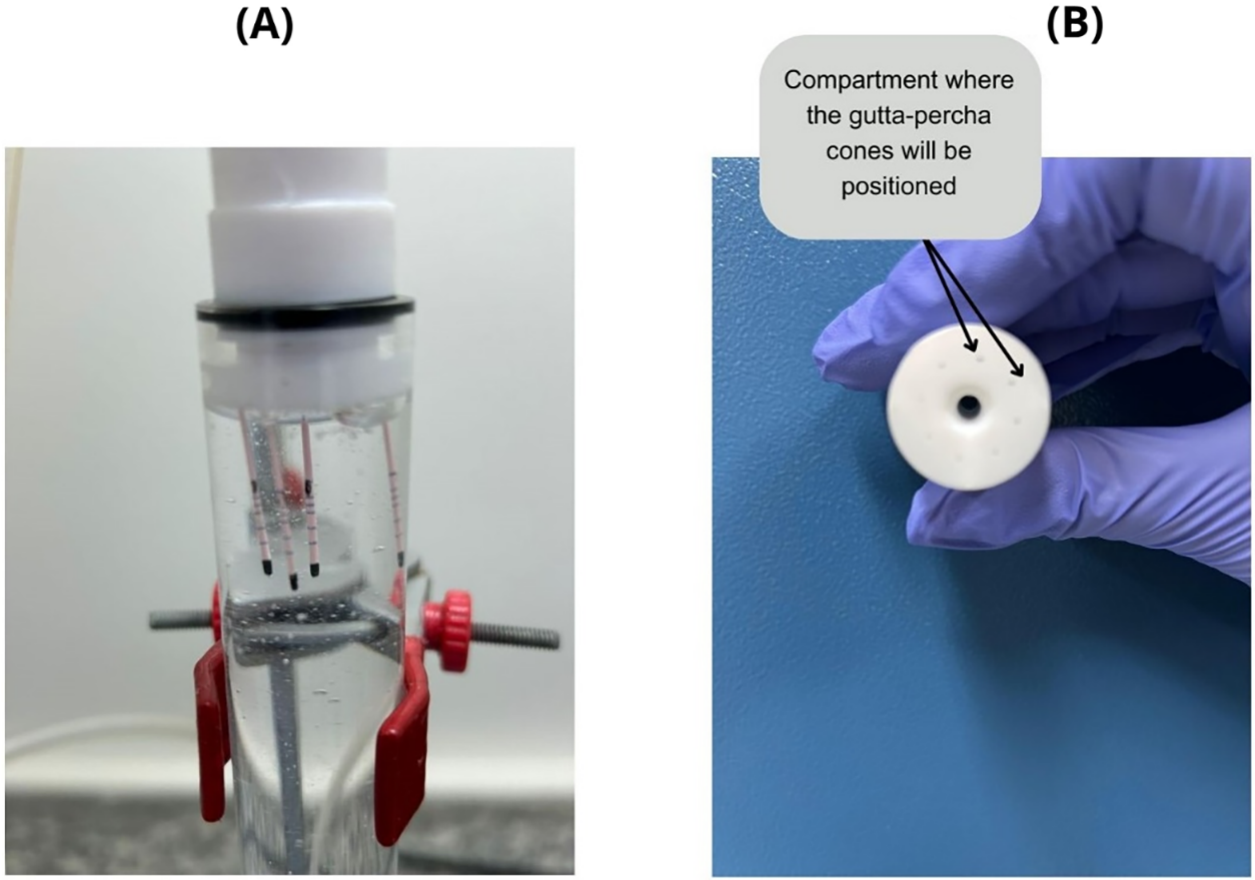
Ozonation procedure for gutta-percha cones. Figure 8A illustrates the positioning of the gutta-percha cones within the chamber where ozonated water flows. Figure 8B shows the component designed to fit into the glass tube, along with details of the perforations used to secure the samples. Source: Author’s own work.

Following ozone exposure, the samples were transferred to screw-cap glass test tubes containing liquid Tryptic Soy Broth (TSB). The tubes were then incubated at 37 °C for 24 h, and microbial growth was assessed based on medium turbidity. In cases where no microbial growth was observed, the tubes remained in incubation for an additional 6 days, totaling a seven-day observation period.

### 5.2 Quantitative Test

Microbial recovery assays were performed to enumerate the colony-forming units (CFU) adhering to the sample surfaces. Following the experimental treatments, each gutta-percha cone was transferred to a 15 mL Falcon tube containing 10 mL of sterile saline solution. The tubes were then manually agitated for 30 min to facilitate the dissociation of the surface-adhered biofilm. From the resulting suspensions, 100 μL aliquots were plated onto Mueller-Hinton Agar using the spread plate technique and incubated at 37 °C for 48 h. This methodological approach was adapted from the protocol established by Taba et al.,^35^ which investigated the inactivation of *S. aureus* on surgical needles using ozone gas under low pressure.

### 5.3 Analysis Using Fourier Transform Infrared Spectroscopy (FTIR)

A chemical analysis was conducted to investigate potential alterations in the composition of gutta-percha cones following ozone exposure. For this purpose, FTIR spectra were obtained in triplicate within the mid-infrared region, encompassing two distinct experimental conditions: untreated gutta-percha cones (in natura) and cones exposed for 7 min to ozonated water within the hydrodynamic system.

FTIR spectroscopy (Spectrum Frontier—PerkinElmer) was performed using a spectrometer equipped with a UATR reflection accessory. Spectra were acquired in the range of 4000 to 550 cm^–1^, with a total of 20 scans and a gain setting of 1. The equipment is housed in a laboratory with controlled temperature and relative humidity conditions.

### 5.4 Scanning Electron Microscopy (SEM)

Scanning electron microscopy was performed under two distinct experimental conditions: on the midsection of untreated (in natura) gutta-percha cones and on cones exposed to ozonated water for 7 min using the hydrodynamic system. Sample fixation was carried out using 2.5% glutaraldehyde. The specimens were stored at temperatures between 2 and 8 °C for 48 h prior to analysis. Subsequently, the samples underwent three 30 min washes using a solution composed of disodium phosphate, monosodium phosphate, and distilled water in a 1:1 ratio. Dehydration was conducted in seven sequential steps using ethanol at increasing concentrations: 30% for 10 min, 50% for 10 min, 70% for 10 min, 90% for 10 min, 90% for 20 min, 100% for 10 min, and finally 100% for 20 min. The samples were then mounted on stubs using double-sided carbon tape and sputter-coated with a thin layer of gold using a Quorum Q150T ES sputter coater. SEM imaging was performed using a Tescan Mira 3 scanning electron microscope.

### 5.5 Optical Profilometry

Surface profilometry was performed using the WYKO NT1100 system. Triplicate samples of untreated (in natura) gutta-percha (GP) cones and cones exposed to ozonated water for 7 min via the hydrodynamic system were prepared. Surface roughness was assessed using the multiple profile method, as described by Nunes et al.^29^

This method proved suitable for evaluating small-scale materials such as gutta-percha cones, allowing for the analysis of multiple profiles within a confined surface region. Axial cross sections were taken from the midsection of the cones, and the primary parameter analyzed was the average roughness (Ra), which represents the arithmetic mean of the absolute height deviations from a central line along the measured profile. Ra is widely used to quantify surface texture and topography.

Topographic analysis was conducted using optical profilometry with the Vision software, and statistical evaluation of the data was performed using GraphPad InStat.

### 5.6 Mechanical Tensile Strength Testing

The mechanical tensile strength of gutta-percha cones was evaluated using a universal testing machine (MTS Criterion, model 42), following the standards established by ISO 527–1:2019–12. For the test, the ends of the cones were secured in the machine’s grips, and a load was applied at a crosshead speed of 1 mm/min until specimen rupture occurred. Throughout the test, the mechanical behavior of the material was recorded via a computer connected to the machine, allowing real-time visualization of the stress–strain diagram. This process revealed two distinct phases in the material’s behavior: the elastic zone, where deformation is reversible, and the plastic zone, characterized by permanent material flow.

A total of 12 gutta-percha samples were used, divided into two groups: six untreated (in natura) and six exposed to the hydrodynamic ozonation system for 7 min. Comparative analysis between the untreated and ozonated groups performed using an unpaired (independent) *t* test, conducted with GraphPad InStat software.
